# 2273 Synthetic cannabinoid usage among psychiatric inpatients

**DOI:** 10.1017/cts.2018.184

**Published:** 2018-11-21

**Authors:** Claire L. Mann, Anahita B. Nia, Sharron Spriggs, Steven Carbonaro, Daniel DeFrancisco, Lyla Parvez, Charles Perkel, Yasmin Hurd

**Affiliations:** 1 Icahn School of Medicine at Mount Sinai; 2 Addiction Institute at Mount Sinai; 3 Mount Sinai Beth Israel

## Abstract

OBJECTIVES/SPECIFIC AIMS: Synthetic cannabinoids (SC) are widely available and are associated with acute psychosis. Our recent study indicated that SC using psychiatric inpatients admitted in 2014 had more psychotic symptoms, aggression, and agitation compared with cannabis [marijuana (MJ)] using patients. The current study will review more charts and will characterize the demographics and presentations of current SC Versus MJ using patients. METHODS/STUDY POPULATION: A chart review was conducted of patients admitted to a New York City inpatient dual diagnosis psychiatric unit from 2014 to 2016. Inclusion criteria were self-reported current SC use or MJ use, or urine toxicology (+) for MJ. RESULTS/ANTICIPATED RESULTS: In total, 585 charts met inclusion criteria, 168 reported current SC use (40 f, 128 m SC users; 122 f, 295 m MJ users). SC using patients were younger (*p*=0.050), more likely to be Black (*p*=0.003), and homeless or living in a shelter (*p*=0.001). SC users were also more likely to be agitated (OR: 2.26) and aggressive (OR: 2.04) and have psychotic symptoms (OR: 3.03) compared with MJ users. SC users received more PRN medication (*p*<0.001) and had longer lengths of stay (*p*=0.001). DISCUSSION/SIGNIFICANCE OF IMPACT: Results demonstrate that current SC users had a different demographic profile compared with current MJ users. Our results also support our previous findings: SC using patients were more likely to be agitated and aggressive and were more likely to demonstrate positive psychotic symptoms.

